# Draft Genome Sequences of Nine New Carnobacterium maltaromaticum Strains Isolated from Diseased Sharks

**DOI:** 10.1128/genomeA.00354-18

**Published:** 2018-05-03

**Authors:** Laura Martinez-Steele, Chris G. Lowe, Mark S. Okihiro, Renaud Berlemont

**Affiliations:** a Department of Biological Sciences, California State University, Long Beach, California, USA; b California Department of Fish and Wildlife, San Diego, California, USA

## Abstract

Here, we report the draft genome sequences of 9 strains of Carnobacterium maltaromaticum (SK_LD1 to SK_LD3 and SK_AV1 to SK_AV6), a member of the *Carnobacteriaceae* family (phylum Firmicutes). These strains were isolated from the brain and the inner ear of three diseased thresher sharks and two diseased salmon sharks. The genome assembly resulted in an average of 3,306,205.9 ± 29,143.9 bp and 3,085 ± 32.67 coding DNA sequences (CDS).

## GENOME ANNOUNCEMENT

Carnobacterium is a lactic acid bacterium that belongs to the family Carnobacteriaceae in the Firmicutes phylum. Of the 10 described species in this genus, Carnobacterium maltaromaticum is the most studied. C. maltaromaticum is used as a preservative agent in the meat and fish industry (1, 2). It is also used in aquaculture for its probiotic properties (3); however, C. maltaromaticum can be pathogenic for fish with reduced immune function (4). In addition, C. maltaromaticum was recently described as a pathogen causing brain and ear infections in young stranded salmon sharks (Lamna ditropis) (5) and common thresher sharks (Alopias vulpinus) (California Department of Fish and Wildlife, unpublished data). Nevertheless, to date, the exact mechanism underlying these infections remains unclear. A recent study suggested that salmon sharks strand in response to a decrease in water temperature due to upwelling events, implying that thermal shock reduces the immune function in young sharks and makes them susceptible to the invasion of opportunistic pathogens (6).

We sequenced the genomes of nine new strains of C. maltaromaticum isolated from the brain and inner ear of 3 stranded thresher sharks and 2 stranded salmon sharks. Infected brains and inner ears were swabbed and plated on Trypticase soy agar (TSA) plates. Individual colonies were isolated, and DNA from the colonies was extracted using the Wizard genomic DNA purification kit (Promega, WI, USA) following manufacturer instructions. DNA extracts were PCR amplified using the primers GM3/GM4 (7) targeting the 16S rRNA bacterial gene, and products were sequenced (Retrogen, San Diego, CA, USA). Retrieved sequences were subject to a BLAST search against the NCBI database and were confirmed as Carnobacterium maltaromaticum (>99% identity).

Next, DNA extracts were sheared using a Covaris S220 system and barcoded and quality controlled by using an Agilent Bioanalyzer and quantitative PCR (qPCR), respectively. Finally, DNA libraries were sequenced on an Illumina HiSeq 4000 platform using paired-end read sequencing runs with 100 cycles in each direction (flow cell identification number HJVGHBBXX) at the University of California, Irvine. Genomes were assembled from the raw Illumina reads using the A5 pipeline (8), and scaffolds of fewer than 3,000 bp were removed from the genome assembly. The final number of scaffolds per genome ranged between 18 and 33, with an average *N*_50_ value of 389,893.2 ± 217.8 bp. JSpeciesWS (9) was used to determine phylogenetic identification of the assembled genomes using the tetranucleotide correlation search (TCS), with all genomes resulting in a Z-score above or in range for Carnobacterium maltaromaticum. Average nucleotide identity (ANId) and tetra correlation analysis (Tetra) between all new genomes resulted in scores above 98% and 0.99, respectively.

The nine draft genomes were uploaded to the National Center of Biotechnology Information (NCBI) and annotated using the Prokaryotic Genome Annotation Pipeline (PGAP) algorithm (10). Annotated genomes had an average length of 3,306,205.9 ± 29,143.9 bp and contained 3,085 ± 32.67 coding DNA sequences (CDS). A detailed analysis of these nine genomes will be performed and published in the near future to identify the genes involved in the virulence mechanisms of this shark pathogen.

### Accession number(s).

The draft genome sequences of the nine Carnobacterium maltaromaticum strains discussed here (SK_LD1 to SK_LD3 and SK_AV1 to SK_AV6) were uploaded to the GenBank database under the genome project accession numbers PKFG00000000, PKFF00000000, PKFE00000000, PKFM00000000, PKFL00000000, PKFK00000000, PKFJ00000000, PKFI00000000, and PKFH00000000, respectively.
